# Supplementary material for: Ultraflexible, cost-effective and scalable polymer-based phase change composites via chemical cross-linking for wearable thermal management
Source: Nat Commun. 2023 Dec 5;14:8060. doi: 10.1038/s41467-023-43772-4 (PMC10698148; doi:10.1038/s41467-023-43772-4)
Supplement: Supplementary file 1 — Supplementary Information [file 41467_2023_43772_MOESM1_ESM.pdf]

Supplementary Information for

Ultraflexible, cost-effective and scalable polymer-based phase change composites via chemical cross-linking for wearable thermal management

Yaoge Jing[1,4], Zhengchuang Zhao[2,4], Xiaoling Cao[1], Qinrong Sun[3], Yanping Yuan[1]*, and Tingxian Li[2]*

[1] School of Mechanical Engineering, Southwest Jiaotong University, Chengdu, 610031, China

[2] Research Center of Solar Power & Refrigeration, School of Mechanical Engineering, Shanghai Jiao Tong University, Shanghai, 200240, China

[3] School of Civil Engineering and Architecture, ChongQing University of Science and Technology, Chongqing, 401331, China

[4] These authors contributed equally: Yaoge Jing and Zhengchuang Zhao

* E-mail: ypyuan@home.swjtu.edu.cn and Litx@sjtu.edu.cn

Content of Supplementary Information

Ultraflexible, cost-effective and scalable polymer-based phase change composites via chemical cross-linking for wearable thermal management 1

1. Preparation method 3

Cross-linking time and temperature (**Fig. S1** and **Table. S1**) 3

Cross-linking chain site and order (**Fig. S2**) 4

Experimental phenomenon (**Fig. S3** and **Fig. S4**) 5

Summary of advantages 7

2. Characterization 8

SEM (**Fig. S5**) 8

AFM (**Fig. S6**) 8

POM (**Fig. S7**) 9

FT-IR (**Fig. S8**) 10

XRD (**Fig. S9**) 11

DSC data (**Fig. S10** and **Table S2**) 12

References comparison (**Table S3**) 13

Compression test (**Fig. S11**) 14

Tensile test of PW@OBC (**Fig. S12**) 15

Tensile test of PW@POE-LDPE (**Fig. S13**) 15

Leakage test (**Fig. S14** and **Fig. S15**) 18

3. Wearable thermal management (**Fig. S16**, **Table S4** and **Fig. S17**) 20

Supplementary References 22

1. Preparation method

Cross-linking time and temperature (Fig. S1 and Table. S1)

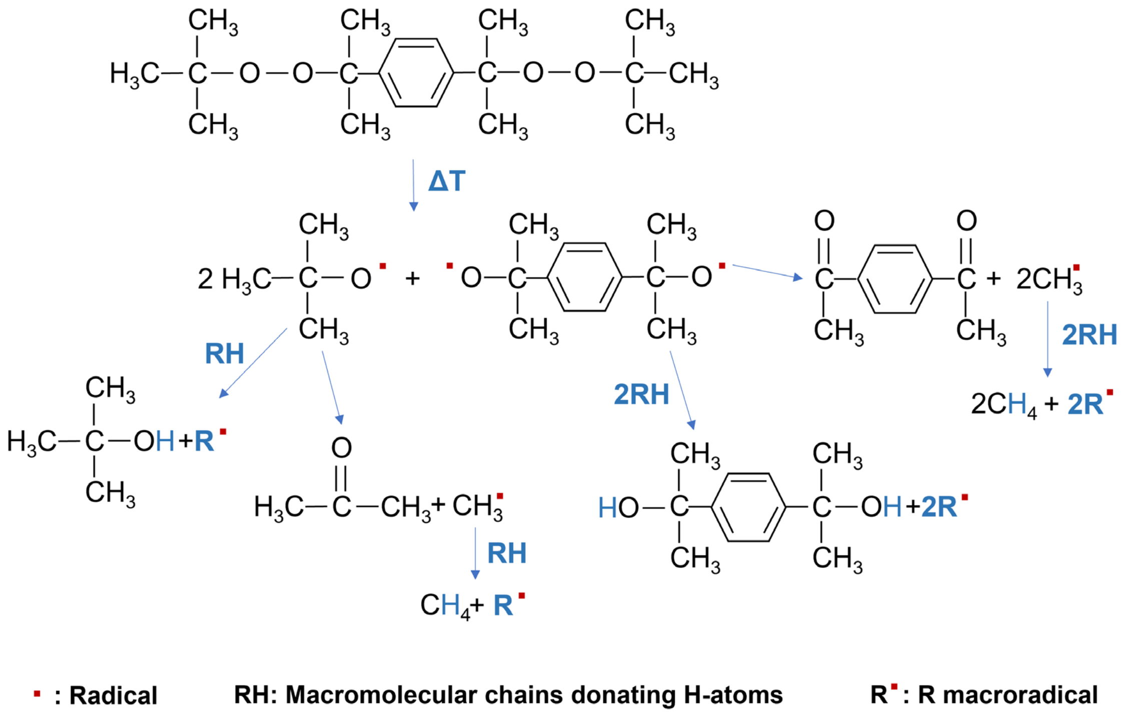

Fig. S1 Decomposition mechanism of Di(tert-butylperoxyisopropyl)benzene[1].

Table S1 Decomposition properties and decomposition products of of Di(tert-butylperoxyisopropyl)benzene[1].

Half-life temperatures for $t_{1/2}$ [a] (°C)	Decomposition products	Relative amount (mol/mol peroxide)	Boiling point (°C)
156 °C for 0.1 h<br>134 °C for 1 h<br>114 °C for 10 h	Tert-butanol	1.83	84.6
	Acetone	0.13	56.5
	Methanes	1.10	-161.5
	Di-(hydroxyl-i-propyl) benzene	0.30	—
	Acetyl hydroxy-i-propyl benzene	0.54	—
	Diacetyl benzene	0.14	120

[a] Data of half-life provided by AkzoNobel Polymer Chemistry.

Fig. S1 shows the decomposition mechanism of Di(tert-butylperoxyisopropyl)benzene, an organic peroxide, also known as an initiator and cross-linking agent, that rapidly decomposes into

volatile organic compounds and free radicals above 156 °C. The rate of cross-linking initiated by the peroxide is determined by its rate of thermal decomposition. Half-life data are essential in selecting the optimal peroxide for specific time/temperature applications. Generally, the half-life time falls by one-third of its value for each 10 °C rise in temperature, and the proposed cross-linking reaction has at least 6 to 10 peroxide decomposition half-lives[2,3].

Table S1 gives this kind of peroxide's half-life and the proportion of decomposition products. Its decomposition products are dominated by the low boiling point of tert-butanol and methanes escaping from the reaction in gaseous form, consistent with the experimental production of bubbles. Meanwhile, it can be deduced that this peroxide decomposes 99.9% of itself after 10 half-lives in 27 min at 176 °C, corresponding to our experimental setting of 180 °C and the complete disappearance of the bubbles in 30 min.

Cross-linking chain site and order (Fig. S2)

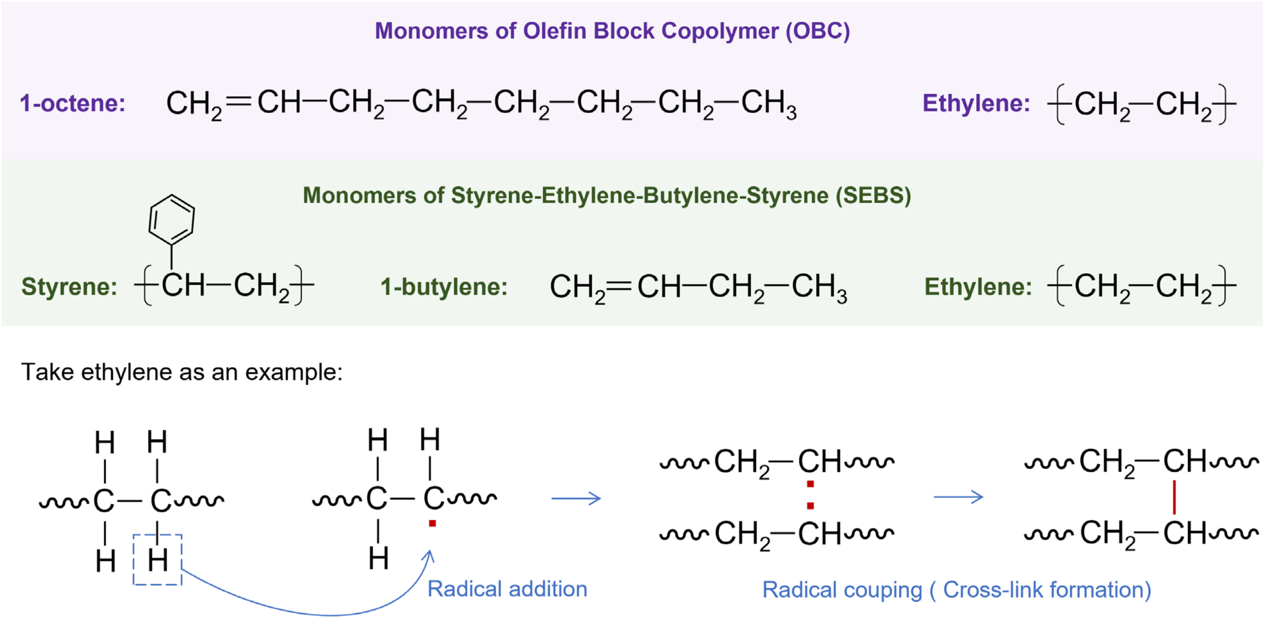

Fig. S2 Specific monomers for OBC cross-linking with SEBS in PW.

The ability of hydrogen atom abstraction depends not only on the type and properties of radical

but also on the structure of the hydrogen donor. The reactivity of distinct unactivated sp^3 C-H bonds, ultimately the site-selectivity of a given reaction, is mainly governed by their bond dissociation energy, polarity and steric accessibility. As a result, C-H bonds usually follow the relative reactivity sequence of benzylic > allylic > tertiary > secondary > primary > vinyl > phenyl. Resonance stabilization of allylic and benzylic structures makes these positions better for hydrogen donors than corresponding alkyl groups[4–6].

The structural monomers of the OBC and SEBS elastomers are given in **Fig. S2**. The specific process of ethylene monomer is an example. Based on the above theories, we infer that all the five monomers mentioned are reactive. Due to the complex factors affecting the cross-linked sites, the exact cross-linking monomers sequence and the mechanism of the sites need to be further investigated.

Experimental phenomenon (Fig. S3 and Fig. S4)

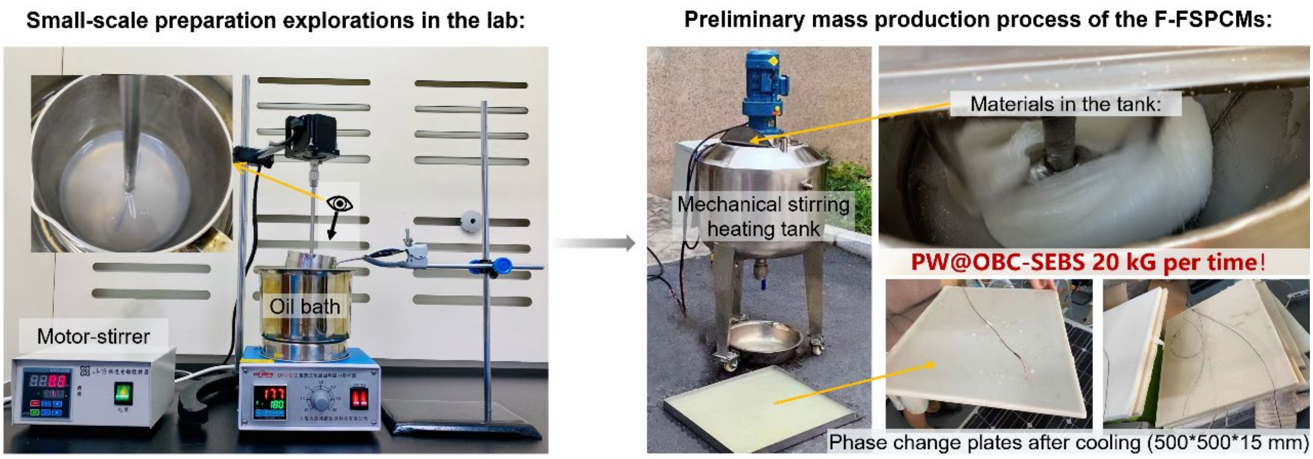

Fig. S3 Schematic diagram of experimental preparation.

Fig. S3 depicts the above laboratory preparation and preliminary mass production process, respectively. We observe fascinating phenomena during the experimental trial. SEBS is first uniformly dispersed in PW, continues in a stiffer white dough state, and finally in a clear gel state. The fine gel

state indicates that PW is well mixed with SEBS, and at this point, with the addition of OBC and peroxide, the system can be mixed quickly and evenly. The feasibility of mass production of the F-FSPCMs is further explored in this study. The same phenomenon can be observed during the fabrication using the mechanical stirring heating tank described above, allowing for rapid preparation of 20 kg in a single run.

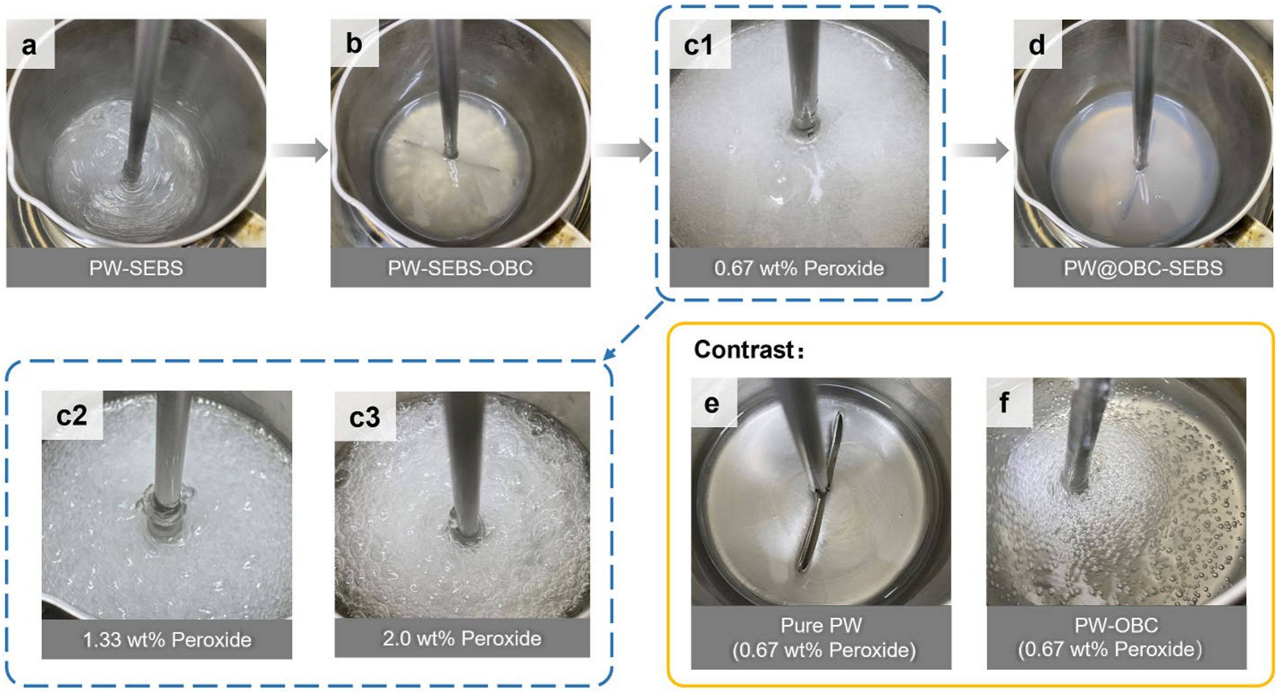

Fig. S4 Experimental phenomena in the material reaction process of F-FSPCMs at 180 °C. a PW-SEBS gel. **b** Further addition of OBC. **c1, c2,** and **c3** Peroxide at different mass contents of 0.67 wt%, 1.33 wt%, 2.0 wt% respectively in the progress of the cross-linking reaction. **d** PW@OBC-SEBS after cross-linking reaction. **e** Pure PW with 0.67 wt% peroxide added. **f** PW-OBC with 0.67 wt% peroxide added.

Fig. S4 gives a more detailed experimental phenomenon of the cross-linking reaction. Dense bubbles are produced immediately when peroxide is added to the physically mixed PW-OBC-SEBS, as shown in **Fig. S4c1**, **c2**, and **c3**. When the peroxide content increases from 0.67 wt% to 2.0 wt%, the reaction is very violent because the bubbles become larger and larger. By comparison, adding

peroxide to pure PW under the same conditions does not produce bubbles (**Fig. S4e**). We assume that PW acts only as a solvent in this reaction. **Fig. S4f** shows the smaller size and number of bubbles after adding equal amounts of peroxide to PW-OBC. The results of subsequent Raman, DSC, and tensile tests also indicate that the OBC undergoes its own cross-linking in PW.

Summary of advantages

The advantages of this solution for mass production can be summarized as follows:

(i) Higher efficiency: In contrast to the one-step feeding method, where melting and mixing processes coincide, the sequential melt blending method used in this work avoids unnecessary mechanical losses that arise due to the high viscosity of the mixture and the sharp increase in the instantaneous resistance of the mixing blades.

(ii) Cheaper equipment: Compared to the preparation process using twin-screw extrusion, the sequential melt blending method can be prepared at atmospheric pressure, and there are no mold restrictions, allowing for the production of sizeable solid phase change plates. The propeller-heated stirring tank in this work costs only 5%-10% of the heavy equipment used in twin-screw extrusion.

(iii) Better results: Physical mixing of PW-OBC-SEBS without adding peroxide requires an extended 1.5-2 h. The longer preparation time allows the impurities in the material to be oxidized at high temperatures, causing the PW-OBC-SEBS to change from white to yellow. In contrast, the chemical cross-linking of PW@OBC-SEBS is signaled by the disappearance of bubbles, and the whole cross-linking process takes only 1 hour, which is faster than physical mixing.

Overall, the innovation of this strategy is based on performance improvement and practical feasibility, which helps to solve the two bottlenecks of phase change materials for thermal management.

2. Characterization

SEM (Fig. S5)

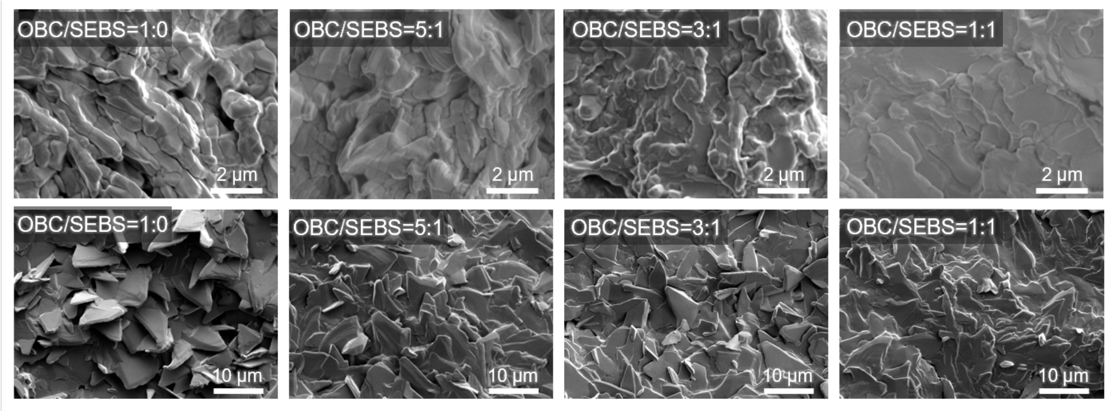

Fig. S5 Scanning electron microscope (SEM) images of liquid nitrogen-treated sections and smooth surfaces after hot pressing of S0-S3, respectively.

AFM (Fig. S6)

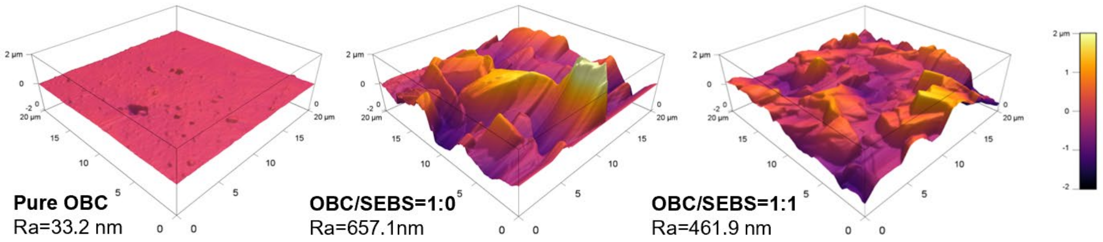

Fig. S6 Atomic force microscope (AFM) surface roughness of OBC, S0, and S3 after hot pressing.

The upper and lower rows of **Fig. S5** show SEM images of the fracture surfaces and smooth surfaces of samples S0-S3, respectively. With the increase of SEBS content, the interfacial compatibility between the three components increases, the voids in the fracture surfaces decrease, and the morphology becomes flatter and more uniform. Correspondingly, the surfaces after hot-pressing also become smoother with the increase of SEBS content and match well with the AFM surface morphology (**Fig. S6**). Among them, the surface of OBC is smooth and dense, with a surface roughness

of 33.2 nm; the surface roughness of PW-OBC is 657.1 nm; and the surface roughness of PW@OBC-SEBS (OBC/SEBS = 1:1) is 461.9 nm. Smooth surfaces with low roughness are beneficial for reducing the contact thermal resistance for material practice. The numerical values of surface roughness presented here are expected to provide a basis for the heat transfer characteristics of surface contact thermal resistance in future F-FSPCMs and electronic devices applications.

POM (Fig. S7)

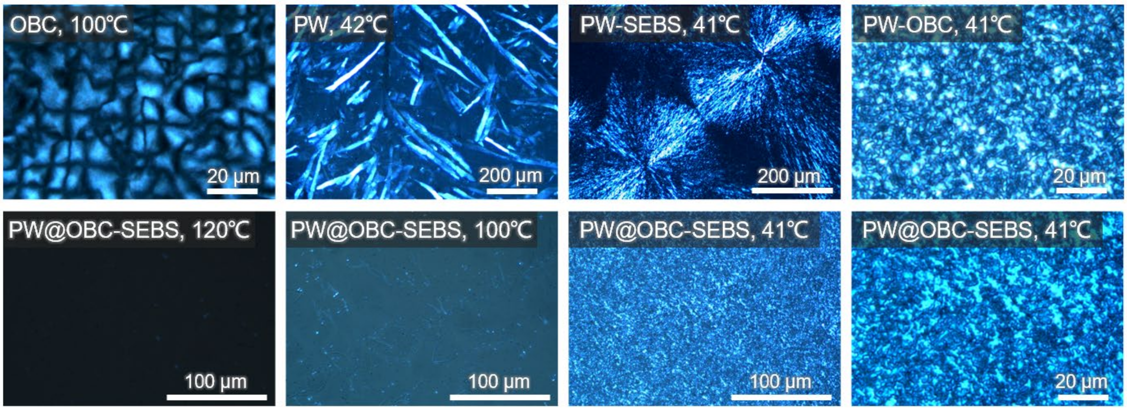

Fig. S7 Polarizing microscope (POM) images of OBC, PW, PW-SEBS, PW-OBC, and PW@OBC-SEBS (S2).

Fig. S7 presents the crystal morphology of F-FSPCMs as observed under a polarizing optical microscope (POM). OBC exhibits maltese cross extinction characteristics during crystallization at 100 °C, with its crystalline region forming small spherical crystals. The isothermal crystallization of PW appears irregular and needle-shaped, with a size ranging from approximately 30-200 μm. When combined with PW, SEBS does not exhibit crystalline behavior but instead forms large maltese cross spherical crystals composed of small needle-shaped paraffin crystals. Previous studies support significant differences in the crystal morphology of PW-SEBS at various ratios. Specifically, at PW content greater than 60 wt%, it forms spherical crystals, with PW preferentially restricted to the EB block[7]. PW-OBC displays a small point-like crystalline form at 41 °C. The cross-linked PW@SEBS-

OBC still exhibits crystalline characteristics at both 100 °C and 41 °C. At 100 °C, OBC appears as tiny bright spots, whereas the PW crystal grains are smaller at 41 °C.

FT-IR (Fig. S8)

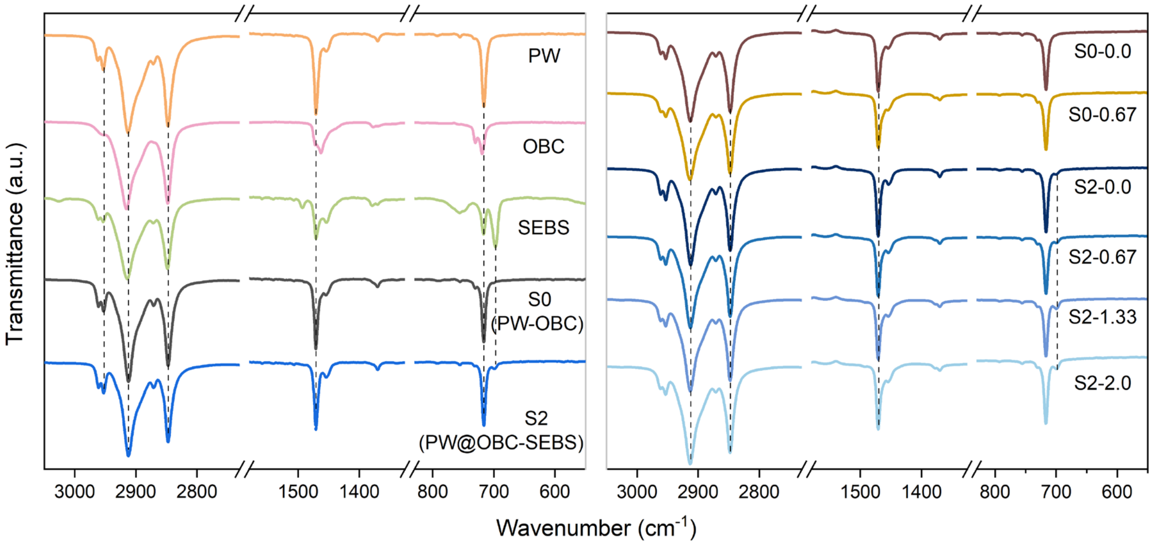

Fig. S8 Fourier transform infrared spectroscopy (FT-IR) results of of PW, OBC, SEBS, S0 with 0 wt% and 0.67 wt% peroxide , and S2 with 0 wt%, 0.67 wt%, 1.33 wt%, and 2.0 wt% peroxide.

The FT-IR spectra results of samples with different peroxide content are shown in **Fig. S8**. The characteristic peaks at 2912 cm^{-1}, 2847 cm^{-1}, 1470 cm^{-1}, and 717 cm^{-1} correspond to the asymmetric and symmetric stretching vibrations of -CH_2 and -CH_3 in PW, as well as the in-plane bending of -$(CH_2)n$ segment in PW. The characteristic peaks at 2920 cm^{-1}, 2847 cm^{-1}, 1470 cm^{-1}, and 698 cm^{-1} correspond to the symmetric stretching vibration, asymmetric stretching vibration, bending vibration, and absorption vibration peaks within the benzene ring in SEBS. For OBC, the main characteristic peak corresponds to the -CH_2 asymmetric stretching vibration over symmetric stretching vibrations at 2920 cm^{-1} and 2850 cm^{-1}. The results indicate that there is no significant shift in the main absorption peaks and no new absorption peaks are generated, suggesting that there is no special chemical interaction between PW and OBC-SEBS blends. However, because of the mild chemical cross-linking

that occurs in PW@OBC-SEBS, it is difficult to determine the degree of cross-linking using infrared spectroscopy.

XRD (Fig. S9)

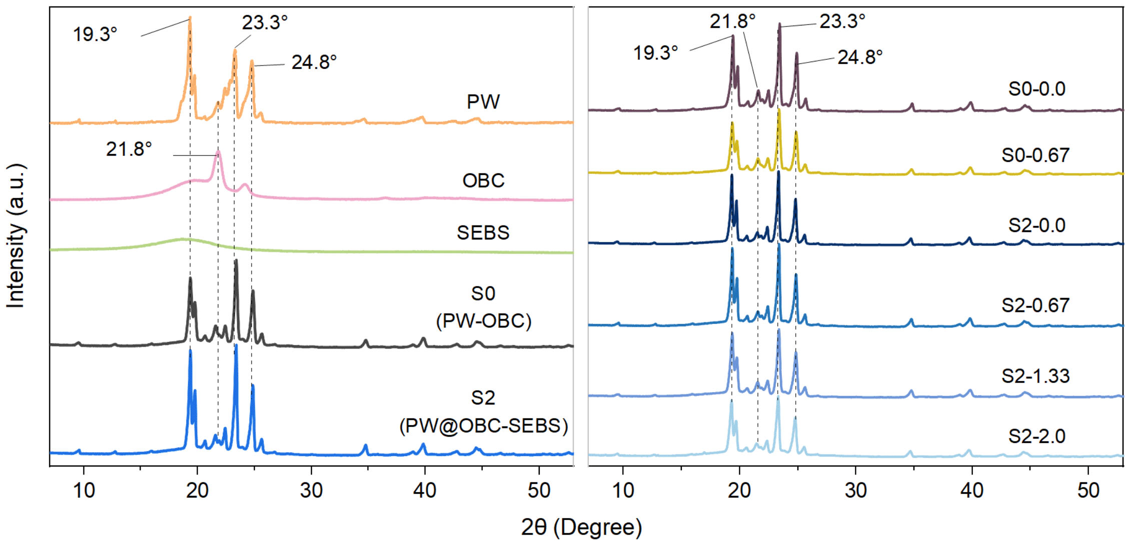

Fig. S9 X-Ray diffraction (XRD) results of of PW, OBC, SEBS, S0 with 0 wt% and 0.67 wt% peroxide , and S2 with 0 wt%, 0.67 wt%, 1.33 wt%, and 2.0 wt% peroxide.

The X-ray diffraction (XRD) results presented in **Fig. S9** reveal distinct patterns for the various materials studied. Specifically, PW exhibits three well-defined diffraction peaks at 19.3°, 23.3°, and 24.8°, which are assigned to its (110), (200), and (210) crystal planes. In contrast, OBC displays a single diffraction peak at 21.8°, indicative of its crystallization. SEBS, on the other hand, shows a broad diffraction peak that suggests its predominantly amorphous state. Notably, the XRD patterns for the F-FSPCMs reveal strong and wide diffraction peaks that correspond to those observed for PW, OBC, and SEBS. Despite this similarity, the crystal shape of PW in the composites remains unchanged, with only a slight reduction in the intensity of the diffraction peak. This behavior can be attributed to the challenges involved in crystallizing PW within the small interstitial gaps between the SEBS-OBC phases.

DSC data (Fig. S10 and Table S2)

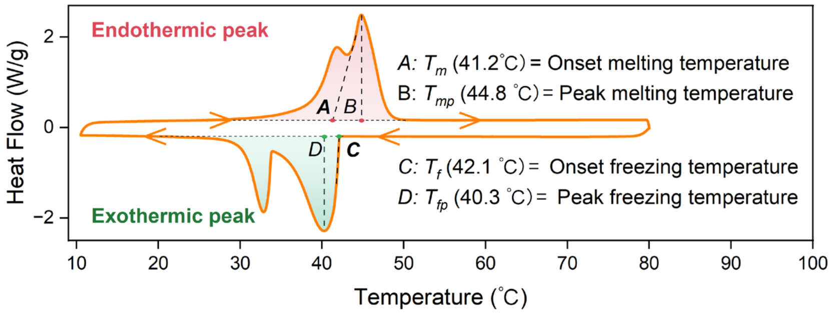

Fig. S10 Definition of phase transition temperature T_m, T_{mp}, T_f, and T_{fp} in DSC.

Table S2 Thermal properties of F-FSPCMs at different ratios.

Samples	Low-Temperature Area						High-Temperature Area			
	T_m (°C)	T_f (°C)	T_{mp} (°C)	T_{fp} (°C)	ΔH_m (J/g)	ΔH_f (J/g)	T_m (°C)	T_f (°C)	ΔH_m (J/g)	ΔH_f (J/g)
PW	41.73	43.23	45.38	41.60	227.8	228.3	—	—	—	—
OBC	—	—	—	—	—	—	123.3	106.5	62.7	60.5
SEBS	—	—	—	—	—	—	—	—	—	—
Theoretical value	41.73	43.23	45.38	41.60	182.2	182.6	123.3	106.5	—	—
PW-0.67	40.76	42.48	44.96	40.64	221.4	220.0	—	—	—	—
S0-0.67	42.53	42.03	44.61	40.48	168.3	168.5	103.2	99.5	9.2	9.4
S1-0.67	41.96	42.03	44.49	40.14	172.9	174.4	103.8	98.9	8.4	7.6
S2-0.67	42.04	41.94	44.82	40.02	172.8	171.7	104.4	98.4	6.2	7.1
S3-0.67	42.03	41.85	44.59	40.34	174.7	176.0	103.2	98.3	5.2	4.8
S0-0.0	42.56	42.06	45.23	39.81	175.5	174.3	104.0	100.0	10.1	10.9
S2-0.0	42.57	42.18	44.98	40.34	177.1	176.0	105.6	99.6	7.4	8.3
S2-0.67	42.04	41.94	44.82	40.02	172.8	171.7	104.4	98.4	6.2	7.1
S2-1.33	41.84	41.59	44.33	40.08	158.7	156.2	104.0	98.5	5.8	6.3
S2-2.0	41.27	41.58	44.12	39.76	158.9	157.5	101.4	97.0	5.6	5.5
**Cycle**	**Accelerated thermal cycle (Sample S2-0.67)**									
After 100	42.23	42.10	44.73	40.27	171.3	174.0	103.8	98.6	6.5	6.8
After 200	41.96	41.78	44.80	39.92	171.7	173.3	103.2	98.6	6.7	6.8
After 300	42.01	41.92	44.90	39.85	172.8	172.5	103.3	98.8	6.9	6.7
After 400	41.89	41.85	44.59	40.01	169.2	170.7	103.1	98.7	7.2	6.9
After 500	41.66	41.82	44.68	40.23	170.2	171.4	103.3	98.9	7.1	6.9

References comparison (Table S3)

Table S3. Comparison of phase change and flexibility properties with those of the corresponding references.

Shaped matrixes	PCM	PCM content (wt%)	T_m/T_f (°C)	ΔH (J/g)	The optimum value of flexibility (Strain at break: %; Strength: Mpa)	Ref.
SEBS	Hexadecane	90	15±1	190±3	Soft gel↔hard gel	8
SEBS/HDPE	Paraffin	75	50.56	151.6	—	9
SEBS/LDPE	Hexadecane	55-80	25.57	106.2-179.8	—	10
SEBS/ASA	Paraffin	10-50	26±2	12.9-74.2	430-740%	11
SBS/TPEE	Paraffin	80	55±2	165.9	60-170%	12
OBC	Paraffin	30-40	47.8±2	47.2-64.4	1500%	13
OBC	Hexadecane	30; 60	9.8; 16.6	45; 148	800-1400%	14
OBC/CNTs	Paraffin	60	46.3	111.7	140-600%	15
OBC/EG	Paraffin	64-85.5	46-49.2	133.7-160.1	3-17%	16
PUA	Myristic acid	60	52.7-55.3	113.4	0.35%; 25-34 Mpa	17
TPEE-EG	Paraffin	60-70	32.2-48.4	112.3-145.9	30-180%; 4.1-7.3 MPa	18
TPU-BN	PEG	60	34.9-61.4	101.2-130.3	44-105%; 5.8-9.3 Mpa	19
PUA	PEG	60	28; 42	113.1	1342.3%; 5 MPa	20
AMD-HDI	PEG	—	38.8-51.1	79.7-116.7	65%; 15 Mpa	21
BQDO-IPDI	PEG	83-89	15-38	54.4-86.1	470-850%; 12-30 MPa	22
PVA-PLA-MXene	PCC	—	35;45	104.5-141.4	37%; 0.5-4 Mpa	23
OBC-SEBS	Paraffin	80	41±1	175±5	23-560%; 0.15-2.7 MPa	This Work

Notes: SEBS (styrene-ethylene-butylene-styrene), EG (expanded graphite), HDPE (high-density polyethylene), LDPE (low-density polyethylene), ASA (acrylonitrile-styrene-acrylate), SBS (styrene-butadiene-styrene), TPEE (thermoplastic ester elastomer), OBC (olefin block copolymer), CNTs (carbon nanotubes), PEG (Polyethylene glycol), TPU (thermoplastic polyurethane), BN (Boron Nitride), PUA (polyurethane acrylate), AMD (4-aminophenyl disulfide), HDI (hexamethylene diisocyanate), BQDO (p-benzoquionone dixoime), IPDI (isophorone diisocyanate), PCC (phase change microcapsule), PVA(polyvinyl alcohol), PLA (polylactic acid).

Compression test (Fig. S11)

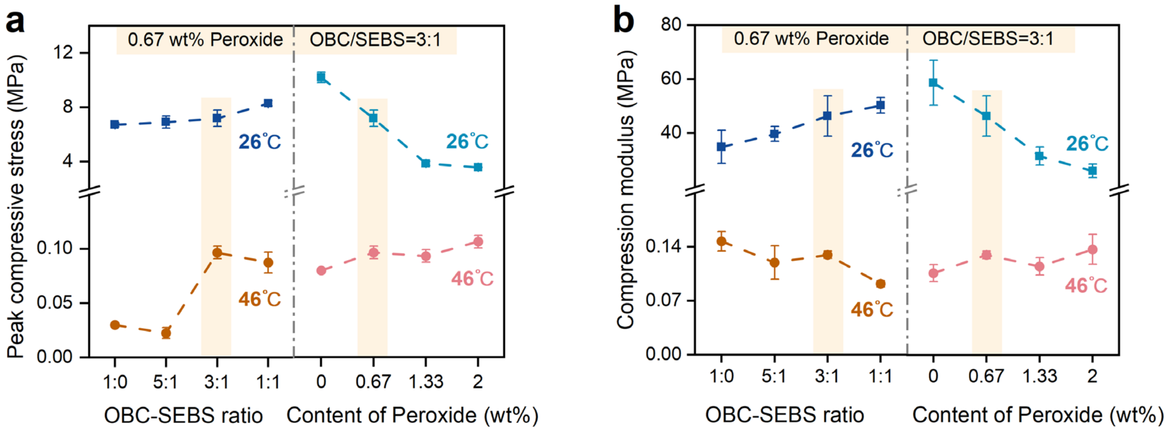

Fig. S11 Compression test results of PW@OBC-SEBS composites (F-FSPCMs). a Influence of OBC-SEBS ratio and peroxide concentration on compression peak stress. **b** Effect of OBC-SEBS ratio and peroxide concentration on compression modulus. Error bars in **a** and **b** are standard deviation (s.d.) from 3 samples.

Tensile test of PW@OBC (Fig. S12)

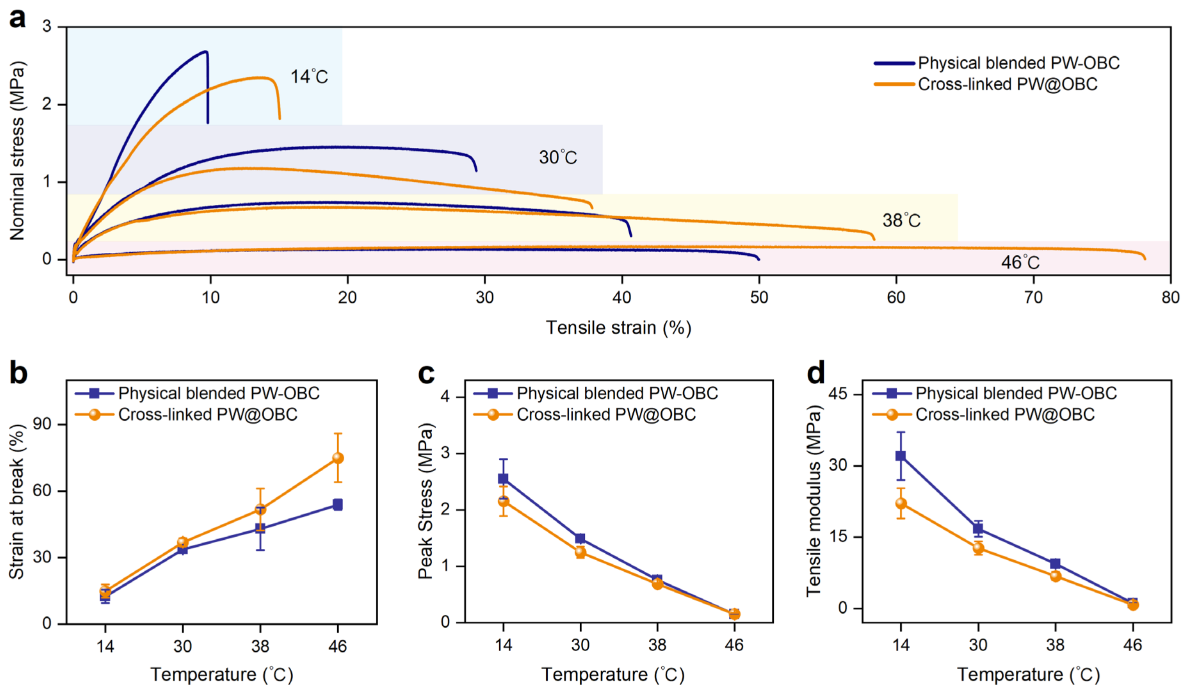

Fig. S12 Tensile test results of physical blended PW-OBC and cross-linked PW@OBC at 14 °C, 30 °C, 38 °C, and 46 °C. a Comparison of strain at break. **b** Comparison of peak stress. **c** Comparison of modulus. Error bars in **b**, **c**, and **d** are standard deviation (s.d.) from 3 samples.

Tensile test of PW@POE-LDPE (Fig. S13)

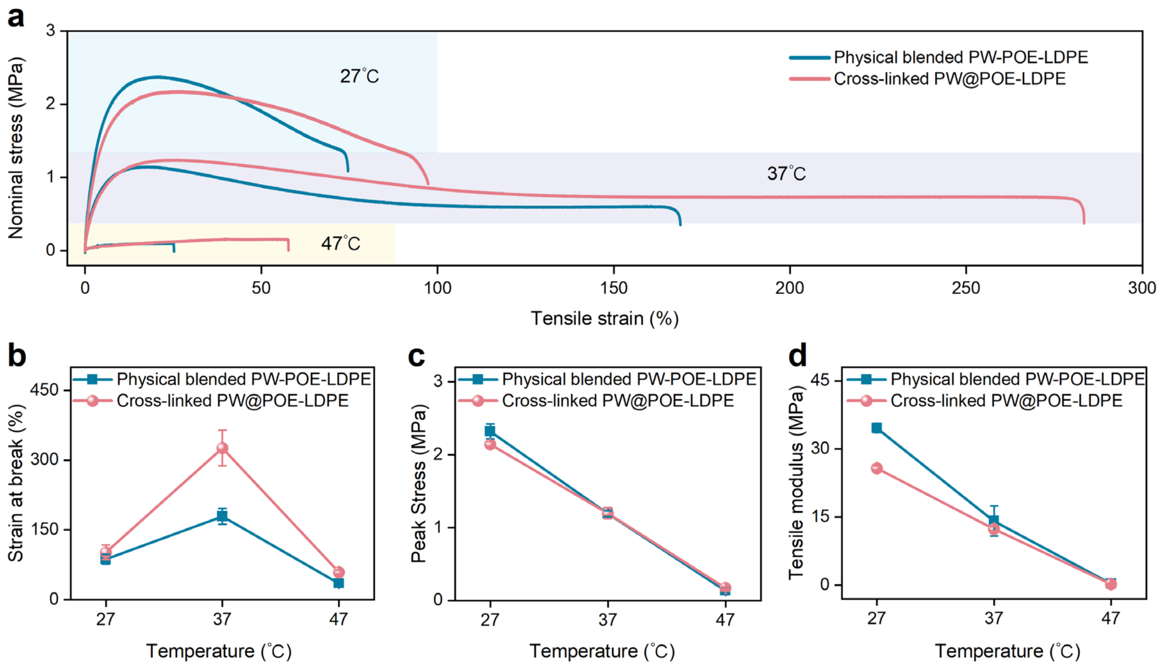

Fig. S13 Tensile test results of physical blended PW-POE-LDPE and cross-linked PW@POE-LDPE at 27 °C, 37 °C and 47 °C. a Comparison of strain at break. **b** Comparison of peak stress. **c** Comparison of modulus. Error bars in **b**, **c**, and **d** are standard deviation (s.d.) from 3 samples.

During our comparative validation experiments, we further employ OBC (20 wt%) and PW (80 wt%) as raw materials, and subject them to both physical blending and chemical crosslinking processes (with peroxide proportion of 0.67 wt%), resulting in two distinct composites, i.e., PW-OBC and PW@OBC, respectively. From the tensile performance results corresponding to four characteristic temperature points (as depicted in **Fig. S12)**, the chemical cross-linked PW@OBC consistently exhibits higher strain at break compared to its physical mixed counterpart. Conversely, there is a marked decrease in its peak stress and modulus.

Meanwhile, the universality tests involving the usage of polyolefin elastomer (POE, ExxonMobil 6202, 12.5 wt%), low-density polyethylene (LDPE, LG MB-9500, 12.5 wt%), and PW (75 wt%) as raw materials have also undergone both physical blending and chemical crosslinking treatment, then resulting in two distinct PW-POE-LDPE and PW@POE-LDPE composites, respectively. As illustrated in **Fig. S13**, a similar pattern emerges, that is, cross-linked specimens exhibit greater strain at break compared to their physical mixed counterparts, while peak stress and modulus display a marginal reduction. However, it is noteworthy that the strain at break rate does not follow a continuous increase with temperature. In the case of PW@POE-LDPE, the strain at break rate is higher at 37 °C compared to that of 47 °C, a performance characteristic that is not initially predicted.

The above results illustrate that the peroxide chemical cross-linking method can render the material "more flexible" by improving strain at break while reducing elastic modulus and peak stress. This enlightens the great potential of chemical cross-linking in transcending the constraints of physical

blending. Not only one or two, but even three or more thermoplastic elastomers can derive synergistic effects. That is to say, engaging other classes of thermoplastic elastomers may lead to more unpredictable performance, which is indeed worthy of in-depth exploration.

Leakage test (Fig. S14 and Fig. S15)

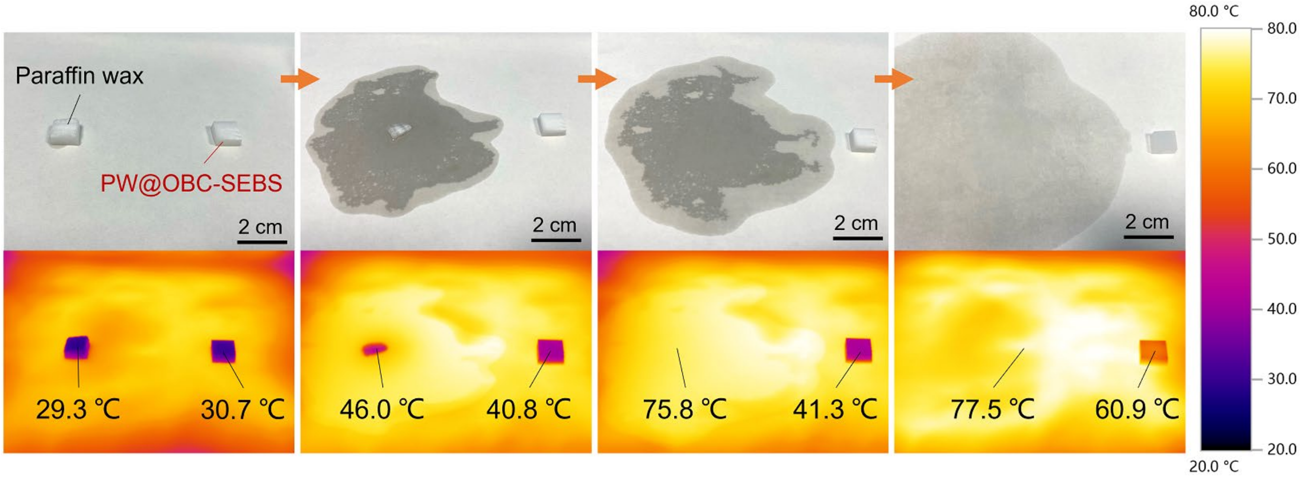

Fig. S14 Leakage comparison diagram of paraffin wax and F-FSPCMs (S2) heated on a hot plate.

The disparity in leakage behavior between paraffin wax (PW) and F-FSPCMs is evident when subjected to an 80 °C hot plate covered with filter paper. PW rapidly undergoes complete liquefaction, forming a distinct puddle of liquid, whereas F-FSPCMs exhibit robust shape retention on the filter paper, displaying no observable indications of leakage.

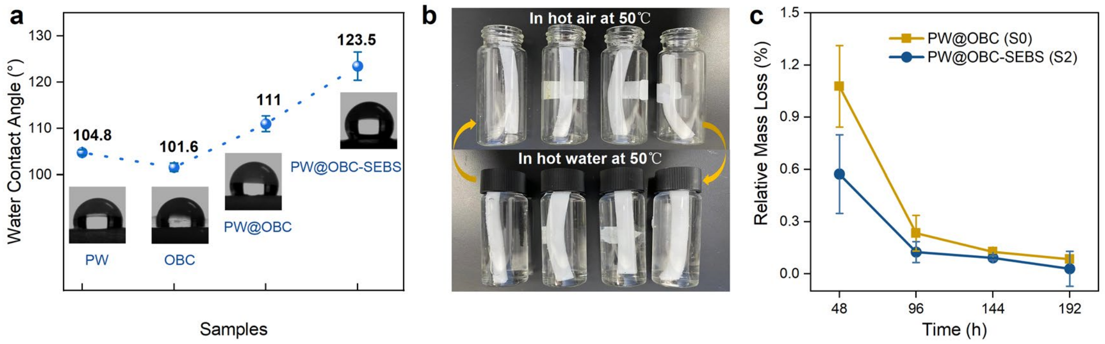

Fig. S15 Destructive leakage testing in water. a Water contact angle results of PW, OBC, PW-OBC, PW@OBC-SEBS (S2). **b** Diagram of the water-air combined leakage test experiment. **c** Relative mass loss curves of PW-OBC and PW@OBC-SEBS. Error bars in **a** and **c** are standard deviation (s.d.) from 3 samples.

The leakage test is conducted by alternating the sample immersion in hot water at 50 °C and drying in an oven at 50 °C for 12 h (one cycle of 24 h), and measuring the mass change after each cycle to evaluate the leak-proof properties of the F-FSPCMs (**Fig. S15b**). The results show that the

relative mass loss decreases with increasing heating time, and there is almost no change in mass loss before and after the third operation (i.e., a total of 144 h of heating) (**Fig. S15c**). The initial mass loss during heating is caused by the melting loss of PW on the surface of F-FSPCMs. Therefore, in the first operation, oil-like floating substances can be observed on the surface of hot water due to the leakage of PW on the surface layer. This phenomenon gradually disappears in subsequent operations as the leakage of PW in the surface layer decreases to trace levels, demonstrating the good leakage-proof and shape-stable properties of PW@OBC-SEBS.

3. Wearable thermal management (Fig. S16, Table S4 and Fig. S17)

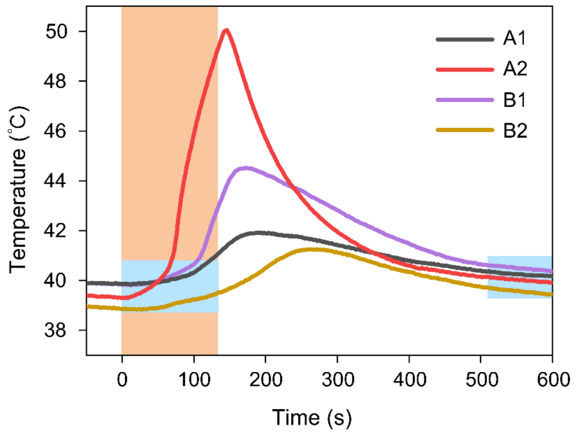

Fig. S16 The reheating-cooling curve at the starting temperature and the phase change temperature (5 mm thickness).

Table S4 Thermal efficiency of F-FSPCMs (S2) temperature control modules.

Module	Thickness (mm)	Mass (g)	ΔH_f (J/g)	Power (W)	Time (s)	Maximum surface temperature (°C)	η (%)
1	3	31.2	174.4	22	900	79.3	27.5
2	5	52.1	174.4	22	900	62.1	45.9
3	7	72.9	174.4	22	900	54.5	64.2
4	7	72.9	174.4	22	800	50.1	72.2
5	7	72.9	174.4	22	600	43.3	96.3
6	7	72.9	174.4	22	400	37.2	144.5

The F-FSPCMs temperature control module developed in this study operates by utilizing the latent heat released during the near-constant temperature phase transition, with the supplied electrical energy serving as the input energy. The thermal efficiency η of the heat storage-release cycle can be defined as:

$$\eta = \frac{m \times \Delta H_f}{P \times t} \times 100\% \quad (1)$$

where m is the mass of the F-FSPCMs (g), ΔH_f is the enthalpy of latent heat (*J/g*), P and t refer to the power of the PI heater band (*W*) and the time to energize the PI heater band (*s*), respectively.

Table S4 presents the calculated thermal efficiency results of 6 groups of measured F-FSPCMs modules. For F-FSPCMs modules 1 to 3, the thickness of the modules increases, resulting in an increase in η from 27.5% to 64.2% for the same heating time, while the maximum surface temperature decreases from 79.3 °C to 54.5 °C. Following the absorption of latent heat, sensible heat absorption begins, leading to a continuous increase in the maximum surface temperature. As sensible heat increases, the thermal efficiency gradually decreases. Modules 3-6 exhibit a decrease in heating time with an increase in η from 64.2% to 144.5%. Notably, module 6's thermal efficiency exceeds the conventional thermal efficiency of less than 100%. This is because the maximum surface temperature of module 6 is 37.2 °C and has not yet reached the phase change temperature of 41 °C, meaning that latent heat storage cannot occur. Thus, Equation (1) is only applicable under conditions where the latent heat process occurs and vice versa.

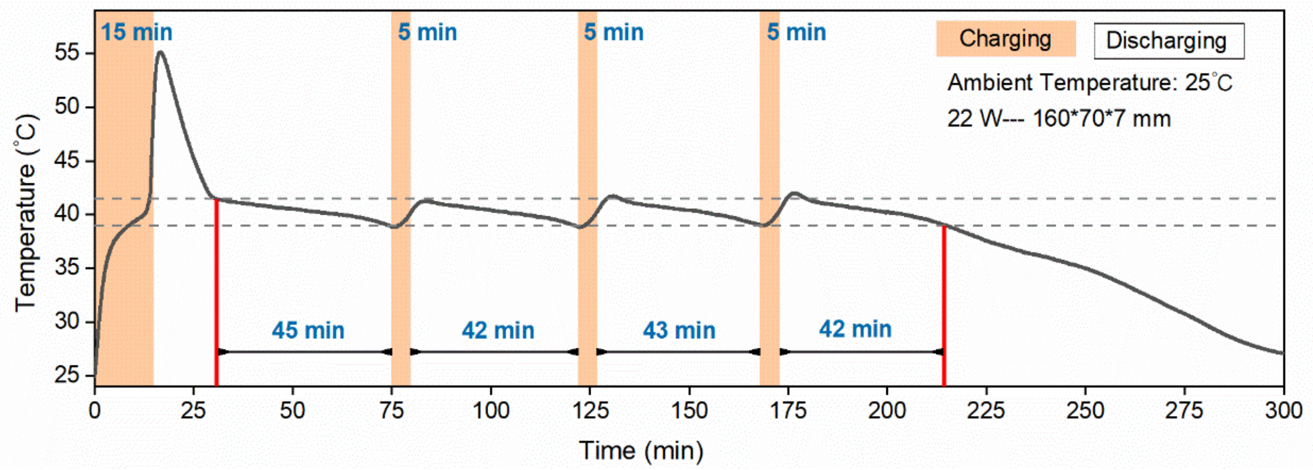

Fig. S17 Temperature control effect of the F-FSPCMs module with intermittently energized heat storage (7 mm thickness).

Supplementary References

1. Thitithammawong, A., Nakason, C., Sabakaro, K. & Noordermeer, J. W. M. Thermoplastic vulcanizates based on epoxidized natural rubber/polypropylene blends: Selection of optimal peroxide type and concentration in relation to mixing conditions. *Eur. Polym. J.* **43**, 4008–4018 (2007).
2. Kruželák, J., Sýkora, R. & Hudec, I. Vulcanization of rubber compounds with peroxide curing systems. *Rubber Chem. Technol.* **90**, 60–88 (2017).
3. Dluzneski, P. R. Peroxide vulcanization of elastomers. *Rubber Chem. Technol.* **74**, 451–492 (2001).
4. Tedder, J. M. The importance of polarity, bond strength and steric effects in determining the site of attack and the rate of free radical substitution in aliphatic compounds. *Tetrahedron* **38**, 313–329 (1982).
5. Lai, W., Li, C., Chen, H. & Shaik, S. Hydrogen-abstraction reactivity patterns from A to Y: the valence bond way. *Angew. Chem. Int. Ed.* **51**, 5556–5578 (2012).
6. Wang, Z. & Wang, F. Radical-mediated selective functionalization of unactivated primary C—H bonds. *Chin. J. Chem.* **40**, 1751–1753 (2022).
7. Zhao, S. & Feng, J. C. Reversible plasticity shape memory effect in sebs/crystallizable paraffin: influence of paraffin content. *Chin. J. Polym. Sci.* **40**, 1697–1705 (2022).
8. Yan, Y., Li, W., Zhu, R., Lin, C. & Hufenus, R. Flexible phase change material fiber: a simple route to thermal energy control textiles. *Materials* **14**, 401 (2021).
9. Chen, P. *et al.* Metal foam embedded in SEBS/paraffin/HDPE form-stable PCMs for thermal energy storage. *Sol. Energy Mater. Sol. Cells* **149**, 60–65 (2016).
10. Chriaa, I. *et al.* Thermal properties of shape-stabilized phase change materials based on Low Density Polyethylene, Hexadecane and SEBS for thermal energy storage. *Appl. Therm. Eng.* **171**, 115072 (2020).
11. Xiang, B., Yang, Z. & Zhang, J. ASA/SEBS/paraffin composites as phase change material for potential cooling and heating applications in building. *Polym. Adv. Technol.* **32**, 420–427 (2021).
12. Huang, Q. *et al.* Pouch lithium battery with a passive thermal management system using form-stable and flexible composite phase change materials. *ACS Appl. Energy Mater.* **4**, 1978–1992

(2021).

13. Zhang, Q., Zhao, Y. & Feng, J. Systematic investigation on shape stability of high-efficiency SEBS/paraffin form-stable phase change materials. *Sol. Energy Mater. Sol. Cells* **118**, 54–60 (2013).
14. Zhang, Q. *et al.* Investigation on the recovery performance of olefin block copolymer/hexadecane form stable phase change materials with shape memory properties. *Sol. Energy Mater. Sol. Cells* **132**, 632–639 (2015).
15. Qi, X., Shao, Y., Wu, H., Yang, J. & Wang, Y. Flexible phase change composite materials with simultaneous light energy storage and light-actuated shape memory capability. *Compos. Sci. Technol.* **181**, 107714 (2019).
16. Wu, S. *et al.* Highly thermally conductive and flexible phase change composites enabled by polymer/graphite nanoplatelet-based dual networks for efficient thermal management. *J. Mater. Chem. A* **8**, 20011–20020 (2020).
17. Li, C., Li, Q., Ge, R. & Lu, X. A novel one-step ultraviolet curing fabrication of myristic acid-resin shape-stabilized composite phase change material for low temperature thermal energy storage. *Chem. Eng. J.* **458**, 141355 (2023).
18. Zhao, X. *et al.* A shape-memory, room-temperature flexible phase change material based on PA/TPEE/EG for battery thermal management. *Chem. Eng. J.* **463**, 142514 (2023).
19. Lin, Y. *et al.* Flexible, highly thermally conductive and electrically insulating phase change materials for advanced thermal management of 5G base stations and thermoelectric generators. *Nano-Micro Lett.* **15**, 31 (2023).
20. Zhang, Q. *et al.* Polyethylene glycol/polyurethane acrylate-based flexible phase- change film with excellent mechanical strength and reversible optical performance. *Energy Fuels* **37**, 3227–3235 (2023).
21. Deng, C. *et al.* Synchronous visual/infrared stealth using an intrinsically flexible self‐healing phase change film. *Adv. Funct. Mater.* **33**, 2212259 (2023).
22. Yang, Y., Cai, X. & Kong, W. A novel intrinsic photothermal and flexible solid-solid phase change materials with super mechanical toughness and multi-recyclability. *Appl. Energy* **332**, 120564

(2023).

23. Li, X. *et al.* Wearable janus-type film with integrated all-season active/passive thermal management, thermal camouflage, and ultra-high electromagnetic shielding efficiency tunable by origami process. *Adv. Funct. Mater.* **33**, 2212776 (2023).
